# Intraoperative defibrillation threshold testing and postoperative long-term efficacy of cardioverter-defibrillator implantation

**DOI:** 10.3892/etm.2012.797

**Published:** 2012-11-05

**Authors:** TIANYI GAN, XIAOZHI CAO, ZHANG YU, BAOPENG TANG, JINXIN LI, GUOJUN XU, XIANHUI ZHOU, YANYI ZHANG, YAODONG LI, JIANGHUA ZHANG

**Affiliations:** 1 Department of Cardiology, First Affiliated Hospital, Xinjiang Medical University, Urumqi, Xinjiang 830054;; 2 Department of Cardiology, Fuzhou General Hospital, Nanjing Command, PLA, Fuzhou 350025, P.R. China

**Keywords:** implantable cardioverter-defibrillator, defibrillation threshold, ventricular arrhythmia

## Abstract

The aim of this study was to determine the defibrillation threshold (DFT) of implantable cardioverter-defibrillators (ICDs) and outcomes of treatment. Sixty-four patients received cardioverter-defibrillator implantation. During implantation, the DFT was determined by the defibrillation safety margin (DSM). All patients were followed up for 12–48 months after the implantation. The overall DFT was 14.27±2.56 J and the DSM was 18.40±1.89 J. Malignant ventricular arrhythmias occurred in 42 patients following cardioverter-defibrillator implantation including 500 episodes of non-sustained ventricular tachycardia (VT) and 289 episodes of persistent VT. VT was treated using antitachycardia pacing (ATP); 265 episodes were treated successfully by a single ATP treatment (91.69%) and 12 episodes were treated successfully by two ATP treatments (4.15%). Twelve episodes were converted by low-energy electrical cardioversion (4.15%). A total of 175 ventricular fibrillation (VF) episodes were identified, of which 18 episodes automatically terminated prior to treatment. In total, 146 episodes were converted by a single cardioversion with a defibrillation energy of 13.21±2.58 J and 11 episodes were converted by two cardioversions with a defibrillation energy of 16.19±2.48 J. It is safe and feasible to determine the DFT by DSM measurement during cardioverterdefibrillator implantation.

## Introduction

Malignant ventricular arrhythmias, including ventricular tachycardia (VT) and ventricular fibrillation (VF), are the main cause of sudden cardiac death (SCD). Many large clinical trials have shown that implantable cardioverter-defibrillators (ICDs) are capable of effectively terminating malignant VT and are the most effective method for preventing SCD. Determination of the defibrillation threshold (DFT) is a critical component of ICD implantation. The traditional method for DFT measurement, though widely used and considered safe, requires frequent VF inductions and may cause severe complications such as cardiogenic shock, cerebral ischemia and death, with an incidence of 0.18–0.39% (1,2). Therefore, some physicians are concerned about the risk of complications related to the induction test and in real world clinical practice, several implant procedures are performed without any induction test. New methods of DFT determination include measurements of the defibrillation safety margin (DSM) (3) and the upper limit of vulnerability (ULV) (4). The DSM method determines a sufficient DSM in 1 or 2 defibrillations, and ensures that the highest ICD defibrillation energy is 10 J more than the DSM. Although this method cannot obtain an actual DFT, it may significantly reduce the frequency of VF or VT episodes. We herein report the results of DFT measurement by the DSM method at the First Affiliated Hospital of Xinjiang. The aim of this study was to observe the long-term outcomes of DFT measurement using the DSM method.

## Patients and methods

### Patients

Patients who underwent ICD implantation at the First Affiliated Hospital of Xinjiang were included in this analysis. Data on patient demographics, clinical history and indication for implantation of the ICD, device type, incidence of shocks, antiarrhythmic medication use and total mortality were collected. Patients of either gender who were older than 18 years of age were eligible for the study if they had experienced at least one or more of the following situations: survival of at least one episode of cardiac arrest (manifested by loss of consciousness) due to ventricular tachyarrhythmia, recurrent, poorly tolerated sustained VT, prior myocardial infarction (MI), congestive heart failure (CHF) due to ischemic or nonischemic causes with left ventricular ejection fraction (LVEF) of ≤35% and (prior to publication of MADIT-II) a documented episode of non-sustained VT, with an inducible ventricular tachyarrhythmia. Patients were excluded from consideration for enrolment if one or more of the following conditions were present: ventricular tachyarrhythmias that potentially had a reversible cause, such as digitalis intoxication, electrolyte imbalance, hypoxia or sepsis, or whose ventricular tachyarrhythmias had a transient cause, such as electrocution or drowning, receiving ICD replacements, life expectancy <2 years due to other medical conditions and inability or refusal to complete the follow-up schedule at the study centre in which the patient was enrolled.

A total of 64 patients, 44 males and 20 females, with a mean age of 65.24±11.35 years, who received ICD implantation at the First Affiliated Hospital of Xinjiang during the period from November 2002 to December 2010 were recruited. Primary diseases included coronary heart disease in 31 patients (48.43%), dilated cardiomyopathy in 21 patients (32.81%), hypertrophic cardiomyopathy in 5 patients (7.81%), right ventricular dysplasia/cardiomyopathy in 1 patient (1.56%) and non-organic heart disease in 6 patients (9.38%). VT or VF was confirmed in all patients by routine ECG, Holter monitor, transesophageal atrial pacing or bedside ECG monitor. Eight patients had concurrent paroxysmal atrial fibrillation (AF), 7 had second degree atrioventricular block (AVB), 7 had sick sinus syndrome and the remaining 42 patients had no other arrhythmic complications (Table I).

### ICD implantation

The left subclavian vein was punctured and then the lead for the right ventricular defibrillation electrode was inserted and advanced to the apex of the right ventricle. For 3-chamber ICDs, the left ventricular electrode was advanced to the lateral cardiac vein or lateral posterior vein. For dual- or 3-chamber ICDs, the right atrial electrode was advanced to the right atrial appendage. The lead for the right ventricular defibrillation electrode was placed at the apex of the right ventricle so that a greater portion of the electrode could deliver electric current to a greater area of the myocardium in order to improve the effectiveness of defibrillation. The electrode threshold, impedance and perceived performance were tested routinely. The ICD systems used are shown in Table II.

### DFT determination

Patients were treated with propofol intravenously for anesthesia. Continuous ECG, blood pressure and blood oxygen monitoring were performed and tracheal intubation and mechanical ventilation were available if required. VT/VF was then induced with short sudden bursts of stimuli or by synchronous electric shock with low-energy (0.6–2 J) T-waves. If the ICD could not accurately and promptly diagnose and terminate VT and VF, external defibrillation was performed immediately. DFT was determined by first using an energy level of 15 J for defibrillation, followed by an increase or decrease of 5 J for a second defibrillation. DSM was defined as the shock energy level that yielded effective defibrillation with the fewest episodes of induced VF (and was considered to be DFT). DSM represented the difference between the highest energy output (30–35 J) of the ICD and the required shock energy level for effective defibrillation, i.e., DSM = highest energy - DFT. DSM was maintained at >10 J and, if ≤10 J, the position of the defibrillation electrode was adjusted and DFT was measured again. The energy level of the ICD for the initial electric shock equaled the DFT, with an increase of 10 J for a second electric shock and was increased to the maximum level for a 3rd–6th electric shock. In the meantime, defibrillation polarity was reversed. All patients were followed up for 12–48 months after the implantation. Our study protocol was approved by the local ethics committee and informed consent to participate in the investigation was obtained from each patient prior to enrolment.

### Follow-up

After implantation, patients were followed up regularly in our device clinic. Shocks were documented by device interrogation and confirmed by an electrophysiologist. Total mortality was assessed by telephone contact.

### Statistical analysis

Continuous variables were summarized as the means ± standard deviation or median and interquartile range (IQR), as appropriate, and categorical variables were summarized as frequencies or percentages.

## Results

### Demographics

A total of 64 patients, 44 males and 20 females, with a mean age of 65.24±11.35 years, who received ICD implantation at the First Affiliated Hospital of Xinjiang during the period from November 2002 to December 2010 were recruited. Primary diseases included coronary heart disease in 31 patients (48.43%), dilated cardiomyopathy in 21 patients (32.81%), hypertrophic cardiomyopathy in 5 patients (7.81%), right ventricular dysplasia/cardiomyopathy in 1 patient (1.56%) and non-organic heart disease in 6 patients (9.38%). VT or VF was confirmed in all patients by routine ECG, Holter monitor, transesophageal atrial pacing or bedside ECG monitor. Eight patients had concurrent paroxysmal AF, 7 had second degree AVB, 7 had sick sinus syndrome and the remaining 42 patients had no other arrhythmic complications (Table I).

### ICD implantation and DFT determination

ICDs were successfully implanted via the venous system in 63 patients. One patient with dilated cardiomyopathy required placement of a left ventricular epicardial electrode via a thoracotomy due to severe deformity of the coronary sinus. The ICD system test results are shown in Table III. The highest energy output of the ICD was 30 J in 51 patients and 35 J in 13 patients. The mean DFT was 14.27±2.56 J and the mean DSM was 18.40±1.89 J. The DSM was 10 J in 1 patient and was increased to >10 J after the defibrillation electrode was repositioned. Hypoventilation occurred in 1 patient during anesthesia induction prior to ICD implantation. After endotracheal intubation and mechanical ventilation, the DFT was successfully measured. All patients emerged from anesthesia quickly after DFT measurement and no complications such as short-term nervous system disorders, vomiting, cardiogenic shock, cerebral ischemia, stroke and death occurred.

### Complications and mortality rate

All patients were followed up for 12–48 months after the implantation. In 1 patient, the right ventricular defibrillation electrode was displaced slightly, which was corrected by a second surgery. There were no other complications such as infection, pocket hematoma, venous thrombosis or broken wires. Three patients died during follow-up, 2 from acute left heart failure and 1 from gastrointestinal bleeding; thus the mortality rate was 4.68%.

### Ventricular arrhythmia events

Data from the cardiac event record reported by the external ICD program-controlled monitor indicated that 42 patients had malignant ventricular arrhythmias, including 500 episodes of non-sustained VT (self-terminated) and 289 episodes of sustained tachycardia (Table IV). Following antitachycardia pacing (ATP), 265 episodes were successfully converted by 1 defibrillation (91.69%), 12 episodes were successfully converted by 2 defibrillations (4.15%) and 12 cardioversions failed (4.15%). These episodes were converted successfully by low-energy electric cardioversion. A total of 175 VF episodes were identified, of which 18 episodes were automatically terminated prior to treatment. Among the VF episodes, 157 episodes were treated by electric cardioversion, of which 146 episodes were successfully converted by 1 cardioversion with a defibrillation energy level of 13.21±2.58 J and 11 episodes were converted by 2 cardioversions with a defibrillation energy level of 16.19±2.48 J.

### ICD electrical storm and inappropriate discharge

There were 3 cases of ICD electrical storm (4.68%). One occurred 15 months after implantation due to electrolyte imbalance and worsening heart failure. Twenty episodes of arrhythmia occurred in 24 h. The condition was gradually controlled following correction of the electrolyte disturbance, treatment with antiarrhythmic drugs, adjustment of ICD parameters and treatment of the primary diseases. VT occurred frequently in another patient who discontinued amiodarone and metoprolol. The condition improved after amiodarone and metoprolol treatment were resumed. In a third patient with right ventricular dysplasia/cardiomyopathy, ICD electrical storm occurred 22 months after the implantation and the cause was unknown. The event could not be controlled by adjusting the ICD parameters and increasing the dosage of antiarrhythmic drugs. It was finally controlled after the patient underwent Ensite3000 3-dimensional mapping and radiofrequency ablation along with administration of amiodarone and metoprolol. In addition, in 6 patients (9.37%) implanted with a single-chamber ICD, AF accompanied by VT was misidentified as VT and VF, leading to inappropriate discharge. After the recognition frequency of VT was increased, heart rate stability and ventricular ECG width were reset and the dose of β-blocker was increased, inappropriate discharge did not recur.

## Discussion

DFT is the minimum energy of ICD discharge required for ventricular defibrillation after VF is induced during ICD implantation, or the minimum energy discharge required for the termination of VF after ICD implantation. The determination of DFT may reduce the energy required for defibrillation and energy output, shorten the charging time, reduce the risk of loss of consciousness, decrease myocardial and cerebral ischemic time and reduce battery consumption. It may also identify electrode problems or myocardial damage, evaluate the integrity of the defibrillation electrode connection and assess the recognition of VT and VF, thus maximizing the benefits of the ICD (5,6).

The major risks of DFT testing are related to DFT test-induced ventricular fibrillation and the electric shock itself. Ventricular fibrillation may stop blood flow and cause relevant complications, including hypoperfusion in the central nervous system, myocardial ischemia and electrical-mechanical separation following defibrillation. Electric shock may cause myocardial damage and dislodgement of thrombi (7). However, these complications are mostly due to repeated VF inductions and electric shocks during DFT measurement using the traditional method. The DFT testing programs include traditional and new DFT measurements. The former includes the gradual reduction, gradual decrease and increase, three-inversion and binary search methods. The latter includes DSM measurement and ULV determination (8,9). In the current study, as determined by the DSM method, the mean DSM was 18.40±1.89 J and mean DFT was 14.27±2.56 J. There were no significant complications during implantation, indicating that this approach is safe and feasible. Hypoventilation occurred in 1 patient during anesthesia induction, which was corrected by endotracheal intubation with mechanical ventilation. Although it did not result in any severe complications, it suggested that sufficient attention should be given to the management of respiratory function.

Studies have shown that the new generation of ICDs using dual-phase wave and activated chassis technology to enhance the effectiveness of defibrillation is of great advantage (10). The application of these technologies may reduce or even eliminate the need for the DFT test (11). Certain researchers also recommended that all patients should be implanted with high-energy output ICDs, without the need for DFT testing. Pires and Johnson (12) retrospectively analyzed 835 patients with ICD implanted during the period from 1996 to 2003 and found that the overall survival rate of the patients without DFT testing was 58%, which was much lower than that of 73% in the patients with DFT testing (P<0.0005). The multivariate analysis showed that DFT testing was an independent risk factor for mortality in patients who received ICD implantation [hazard ratio (HR), 2.031, 95%; confidence interval (CI), 1.253–3.290; P=0.004]. In the current study, follow-up results showed that the ICD was able to successfully identify and defibrillate VF. The discharge energy was 13.21±2.58 J in the first defibrillation and 16.19±2.48 J in the second defibrillation, indicating that the defibrillation energy of the DFT setting determined by the DSM method could not only minimize the energy required for defibrillation, but also effectively convert VF.

In DFT testing, an excessively high DFT is a potential risk. Therefore, the DSM should be maintained at >10 J. Russo *et al*(13) reported that the probability of an excessively high DFT was >6% in a DFT test. Once the DFT is excessively high, action should be taken to adjust it, including changing the polarity of defibrillation, adjusting the position of the defibrillation electrodes, increasing the number of defibrillation electrodes, such as by using a subcutaneous meshed electrode or arrayed electrode and changing the slope of the defibrillation wave (14,15). In the current study, the DSM was only 10 J in 1 patient. We changed the polarity of the defibrillation shock first, but this was not effective. We then adjusted the position of the defibrillation electrode so that the DSM was >10 J, which fulfilled the implantation requirement.

ICD electrical storm refers to >2 episodes of VT or VF within 24 h which require ICD intervention (16). Early antiarrhythmics versus implantable defibrillators (AVID) studies suggested that electrical storm was an independent risk factor of death, with an incidence of 20% outside of China (17) and 10–17% in China (18,19). In the current study, electrical storm occurred in 3 patients, thus the incidence was 4.68%. An electrical storm is likely to cause frequent ICD discharges, thus causing discomfort and psychological symptoms such as anxiety and fear. Additionally, frequent ICD discharges will shorten the battery life. When ICD electrical storm occurs, the first step of treatment is to eliminate the causes, followed by positive comprehensive management, appropriate ICD adjustment and radiofrequency ablation when necessary (20). Misidentification of VT and VF and inappropriate discharge usually occur in patients implanted with single-chamber ICD and particularly in the case of supraventricular tachyarrhythmias, with a reported incidence of 20% (21). We found a misidentification rate of 9.37% in patients with a single-chamber ICD in which atrial fibrillation with ventricular tachycardia was misidentified as VF, resulting in inappropriate discharges. In such cases, comprehensive treatment including increasing the identifying frequency of ventricular tachycardia, resetting the heart rate stability and ECG width and increasing the dosage of metoprolol should be used to reduce or eliminate the false identification and inappropriate ICD discharges.

In conclusion, our study suggests that the DSM is a safe and feasible approach for determining the DFT during ICD implantation. The intensity of the first shock may be diminished by this method. Furthermore, the defibrillation energy determined by this method was able to convert VF effectively. Therefore, the DSM should be conserved in the long-term.

## Figures and Tables

**Table I:** Patient characteristics (n=64).

Clinical characteristic	
Gender	
Male	44 (68.75%)
Female	20 (31.25%)
Age (years)	65.24±11.35
Left ventricular ejection fraction	0.38±0.19
Heart disease	
Coronary artery disease	31 (48.30%)
Dilated cardiomyopathy	21 (32.81%)
Hypertrophic cardiomyopathy	5 (7.81% )
Right ventricular dysplasia/cardiomyopathy	1 (1.56%)
Non-organic heart disease	6 (9.38% )
NYHA cardiac function	
I–II	38 (59.37%)
III–IV	26 (40.63%)
Arrhythmia	
Ventricular fibrillation	29 (45.31%)
Sustained ventricular tachycardia	19 (29.68%)
Non-sustained ventricular tachycardia	16 (25.00%)
Concurrent arrhythmia	
Atrial fibrillation	8 (12.50%)
2nd degree atrioventricular block	7 (10.94%)
Sick sinus syndrome	7 (10.94%)

Data are presented as the means ± standard deviation or no. (percentage).

**Table II:** Implantable cardioverter-defibrillator (ICD) systems implanted.

ICD system	No. of cases
Medtronic ICD	42
Single-chamber ICD	18
Micro Jewel 7221	6
Marquis 7230	10
Virtuoso D164VWC	1
GEM 7227	1
Dual-chamber ICD	17
GEM DR 7271	1
Intrinsic 7288	2
Marquis 7274	13
Virtuoso D164AWG	1
3-chamber ICD	7
Insync III Marquis 7279	2
Concerto C174AWK	1
St. Jude ICD	22
Single-chamber ICD (Epic V-196)	9
Dual-chamber ICD (Epic V-239)	9
3-chamber ICD (Epic V-350)	4

**Table III:** ICD system test results.

Parameters of ICD system test	Result (mean ± SD)
Atrial pacing threshold (V)	0.70±0.31
Atrial electrode impedance (Ω)	661±142.35
P wave amplitude (mV)	3.67±2.32
Ventricular pacing threshold (V)	0.78±0.26
Ventricular electrode impedance (Ω)	771±121.22
R wave amplitude (mV)	7.8±2.41
DFT (J)	14.27±2.56
DSM (J)	18.40±1.89

Data are presented as the mean ± standard deviation. ICD, implantable cardioverter-defibrillator; DFT, defibrillation threshold; DSM, defibrillation safety margin.

**Table IV:** Ventricular arrhythmias and ICD treatment during follow-up.

			ATP treatment			Electric defibrillation	
Ventricular arrhythmia	No. of episodes	Spontaneous resolution	Once	Twice	Low-energy electrical cardioversion	Once	Twice
Non-sustained VT (episode)	500	500	0	0	0	0	0
Sustained VT	289	0	265 (91.69%)	12 (4.15%)	12 (4.15%)	0	0
VF	175	18	0	0	0	146	11
Defibrillation energy (J)						13.21±2.58	16.19±2.48

Data are presented as the means ± standard deviation or number (percentage). ICD, implantable cardioverter-defibrillator; ATP, antitachycardia pacing; VT, ventricular tachycardia; VF, ventricular fibrillation.
